# Circular RNA circMDM2 accelerates the glycolysis of oral squamous cell carcinoma by targeting miR‐532‐3p/HK2

**DOI:** 10.1111/jcmm.15380

**Published:** 2020-05-15

**Authors:** Zhao Zheng, Xiaozhou Ma, Hongfa Li

**Affiliations:** ^1^ The School and Hospital of Stomatology Tianjin Medical University Tianjin P.R. China

**Keywords:** circMDM2, glycolysis, HK2, oral squamous cell carcinoma

## Abstract

Circular RNAs (circRNAs) function as an essential regulator in the progression of oral squamous cell carcinoma (OSCC). However, the potential roles and mechanism of circRNAs in OSCC are still elusive. Here, this research investigates the roles and molecular mechanism of novel circRNA (circMDM2) in OSCC progression. Clinically, circMDM2 was overexpressed in OSCC tissue and cells, and the overexpression served as a poor prognostic factor for OSCC patients. Functionally, cellular experiments confirmed that circMDM2 accelerated OSCC cell proliferation and glycolysis in vitro and circMDM2 knockdown repressed the tumour growth in vivo. Mechanistically, circMDM2 sponged miR‐532‐3p to promote the hexokinase 2 (HK2), forming the circMDM2/miR‐532‐3p/HK2 axis. In conclusion, these findings demonstrated that circMDM2/miR‐532‐3p/HK2 axis promotes the proliferation and glycolysis of OSCC, rendering a potential diagnostic biomarker and prospective therapeutic target for OSCC.

## 1 INTRODUCTION

Oral squamous cell carcinoma (OSCC) acts as one of the most common cancers of oral or head and neck region with high degree of malignancy.^1^, ^2^ Despite the breakthroughs in the clinical diagnosis and therapeutic strategy, the morbidity and mortality rates of OSCC are still increasing yearly.^3^, ^4^ Moreover, the overall five‐year survival rate of OSCC patients remains poor due to tumour recurrence and metastasis with a high frequency.^5^ Among the carcinogenic factor, genetic or epigenetic factors are increasing remarkably.^6^ Therefore, the investigation of genetic‐related pathogen is urgent for OSCC diagnosis and treatment.

Circular RNAs (circRNAs) are large portion of noncoding transcripts characterized by covalently closed loop.^7^, ^8^, ^9^ Abundant quantity of circRNAs is identified by next‐generation sequencing techniques.^10^, ^11^, ^12^ Although being lack of protein coding potential, circRNAs wildly participate in the physiopathologic progression of human cancers, including OSCC.^13^ For example, hsa_circ_0072387 expression in OSCC is significantly down‐regulated, acting as a valuable predictor for OSCC and being associated with the TNM stage in OSCC.^14^ CircRNA hsa_circ_0005379 level is significantly lower in OSCC tissue compared to paired tissue, and hsa_circ_0005379 overexpression effectively inhibits the migration and proliferation of OSCC cells involving in the regulation of the epidermal growth factor receptor (EGFR) pathway.^15^ Thus, the critical roles of circRNAs in the OSCC are more and more significant.

In present research, we performed the investigation for the expression and functions of circMDM2 in OSCC tumorigenesis proliferation and glycolysis. Here, we found that circMDM2 was up‐regulated in the OSCC tissue and cell lines. Moreover, circMDM2 functioned as a critical regulator for the OSCC proliferation and glycolysis through circMDM2/miR‐532‐3p/HK2 axis.

## 2 MATERIALS AND METHODS

### 2.1 Tissue specimens

Total 20 OSCC tumour samples and paired adjacent normal tissues were obtained from surgery. None of these patients received any chemoradiotherapy. The clinical specimen collection was approved by the Ethics Committee of Stomatology of Tianjin Medical University. Informed consent was obtained from each patient. After tissue removal from the operation, these specimens were immediately snap‐frozen in −80°C until ready for use.

### 2.2 Cells culture and transfection

The OSCC cell lines (SCC25, CAL27) and normal oral keratinocytes (NHOK) were purchased by ATCC (American Type Culture Collection) and Institute of Biochemistry and Cell Biology of the Chinese Academy of Sciences. Cells were cultured in DMEM (Dulbecco's modified Eagle's medium; Gibco) medium supplemented with 10% foetal bovine serum (FBS; Gibco) 100 U/mL penicillin and 100 mg/mL streptomycin. Specific short hairpin RNAs (shRNAs) targeting circMDM2 were synthesized by GenePharma. OSCC cells (SCC25) were seeded on 6‐well plates and transfected with specific shRNA (100 nM) or control shRNA (100 nM) using Lipofectamine 2000 (Invitrogen) following the manufacturer's protocol. The primer sequences of shRNA sequences are presented in Table S1.

### 2.3 RNA extraction and real‐time qPCR

Real‐time qPCR was performed as previously described.^9^ Total RNA was extracted from OSCC tissues and cells using TRIzol reagent (Invitrogen) according to the manufacturer's instruction. RNA was reverse transcribed into cDNA using PrimerScript RT Master Mix (Takara). For circRNA and mRNAs expression, the qRT‐PCR was determined using QuantiTect SYBR Green PCR Kit (Qiagen) using β‐actin internal control. For miRNA expression, PCR was determined using miRNA qRT‐PCR Starter kit (Riobo) using U6 internal control. The qRT‐PCR was performed using ABI Prism 7500 Fast real‐time PCR system. Relative RNA expression was calculated by 2^−ΔΔCt^ method normalized to beta‐actin. The primer sequences for PCR are described in Table S1.

### 2.4 Actinomycin D and RNase R assay

For actinomycin D (Act D) assay, RNA was added with 2 mg/mL Act D (Sigma) and the circular and linear RNA expression levels were analysed by using of a qRT‐PCR assay. For RNase R assay, total RNAs were isolated from cells and then incubated with RNase R (5 U/mg; Epicentre Technologies) using RNeasy MinElute Cleaning Kit (Qiagen). Subsequently, RNA transcripts were analysed by RT‐PCR.

### 2.5 Cellular CCK‐8 proliferation

Cellular viability assay was measured using Cell Counting Kit‐8 (CCK‐8). Cells (1 × 10^3^ per well) were plated into 96‐well plate with culture medium. CCK‐8 were added to each well for 4 hours. The spectrometric absorbance was measured by microplate reader (Bio‐199 Rad) at 450 nm. Each experiment was performed three times.

### 2.6 Ethynyl‐2‐deoxyuridine (EdU) incorporation assay

Cell proliferative viability EdU assay was performed according to instructions. Briefly, OSCC cells (5 × 10^3^ cells per well) were transfected with plasmids or shRNA, and then, EdU (50 μM, RiboBio) labelling medium was added for incubation for 2 hours under 5% CO_2_ at 37°C. Cells were incubated with 100 μL of Hoechst 33342 solution for 30 minutes at room temperature in the dark and then washed with 100 μL of PBS. The observation was performed under a fluorescent microscope, and the percentage of EdU‐positive cells was calculated.

### 2.7 Western blot analysis

The cellular protein was collected by RIPA lysis buffer (KeyGEN). Protein (20 μg) was electrophoresed in SDS‐PAGE gels (10%) and then transferred to polyvinylidene difluoride (PVDF) membranes (Millipore). The PVDF membranes were incubated with primary antibodies at 4°C overnight, including anti‐HK2 (ab104836, 1:1000 dilution; Abcam). Then, PVDF membranes were treated with beta‐actin at 37°C for 2 hours. Bands were measured using ECL Western Blot Kit (Thermo Fisher Scientific) on the Lab Works (Bio Imaging Systems).

### 2.8 Glucose consumption, lactate production and ATP level

Glucose consumption, lactate production and ATP level were, respectively, detected using Glucose Assay Kit (Sigma), Lactate Colorimetric/Fluorometric Assay Kit (BioVision) and CellTiter‐Glo Luminescent Cell Viability Assay (Promega) according to the instructions.

### 2.9 Extracellular acidification rate assay

Extracellular acidification rate (ECAR) was performed as previously reported.^16^ For ECAR, glucose (10 mM), the oxidative phosphorylation inhibitor oligomycin (1.0 μM) and the glycolytic inhibitor 2‐deoxyglucose (2‐DG, 50 mM) were orderly administrated at indicated time points.

### 2.10 Subcellular fractionation location

Cytoplasmic and nuclear RNA in OSCC cells were isolated and purified using the PARIS Kit (Life Technologies) according to the manufacturers' instructions.

### 2.11 Fluorescence in situ hybridization

Hybridization for the subcellular location was performed as previously reported.^17^ The isolation was incubated with circMDM2 and miR‐532‐3p probes. Images were captured using a Nikon inverted fluorescence microscope.

### 2.12 Luciferase reporter assay

Dual‐Luciferase expression vectors (pmirGLO) were purchased from GenePharma Co. Ltd. circMDM2 and HK2 3'‐UTR sequences were generated by PCR amplification and then subcloned into the luciferase reporter vector pGL3‐basic (Promega). Wild‐type and mutant of plasmids were generated by PCR reaction. The luciferase assays were performed using a Luciferase Assay Kit (Promega) according to the manufacturer's protocol using the Dual‐Luciferase Reporter Assay System (Promega). The relative expression was calculated based on firefly luciferase module normalized to Renilla luciferase activity in triplicates and was independently repeated three times. Data were shown as the mean value ± *SD*.

### 2.13 Xenograft mice model in vivo

Male BALB/c nude mice (6 weeks old, 10 mice) were purchased from Slac Laboratory Animal Center. Animal experiments were approved by the Animal Care and Use committee of The School and Hospital of Stomatology of Tianjin Medical University. Experiments details were carried out according to the Guide for the Care and Use of Laboratory Animals and in compliance with the institutional ethical guidelines. Approximate 5 × 10^6^ stable SCC25 cell lines with circMDM2 shRNA transfection were injected into flak of nude mice. Tumour volume was calculated by (length × width^2^ × 0.5). Mice were killed after 4 weeks, and neoplasms were weighted.

### 2.14 Statistical analysis

SPSS 19.0 software (SPSS) and GraphPad Prism version 7.0 were used to analyse the experimental data. Data were presented as means ± standard deviation (*SD*). Student's *t* test was used to compare the significant difference of two groups, and one‐way analysis of variance (ANOVA) was used to compare the significant difference of multiple group comparisons. The Kaplan‐Meier method with log‐rank test was used to analyse OSCC patients' survival curve. A *P*‐value < 0.05 was considered statistically significant.

## 3 RESULTS

### 3.1 CircRNA circMDM2 overexpression indicates the poor prognosis of OSCC patients

**FIGURE 1:**
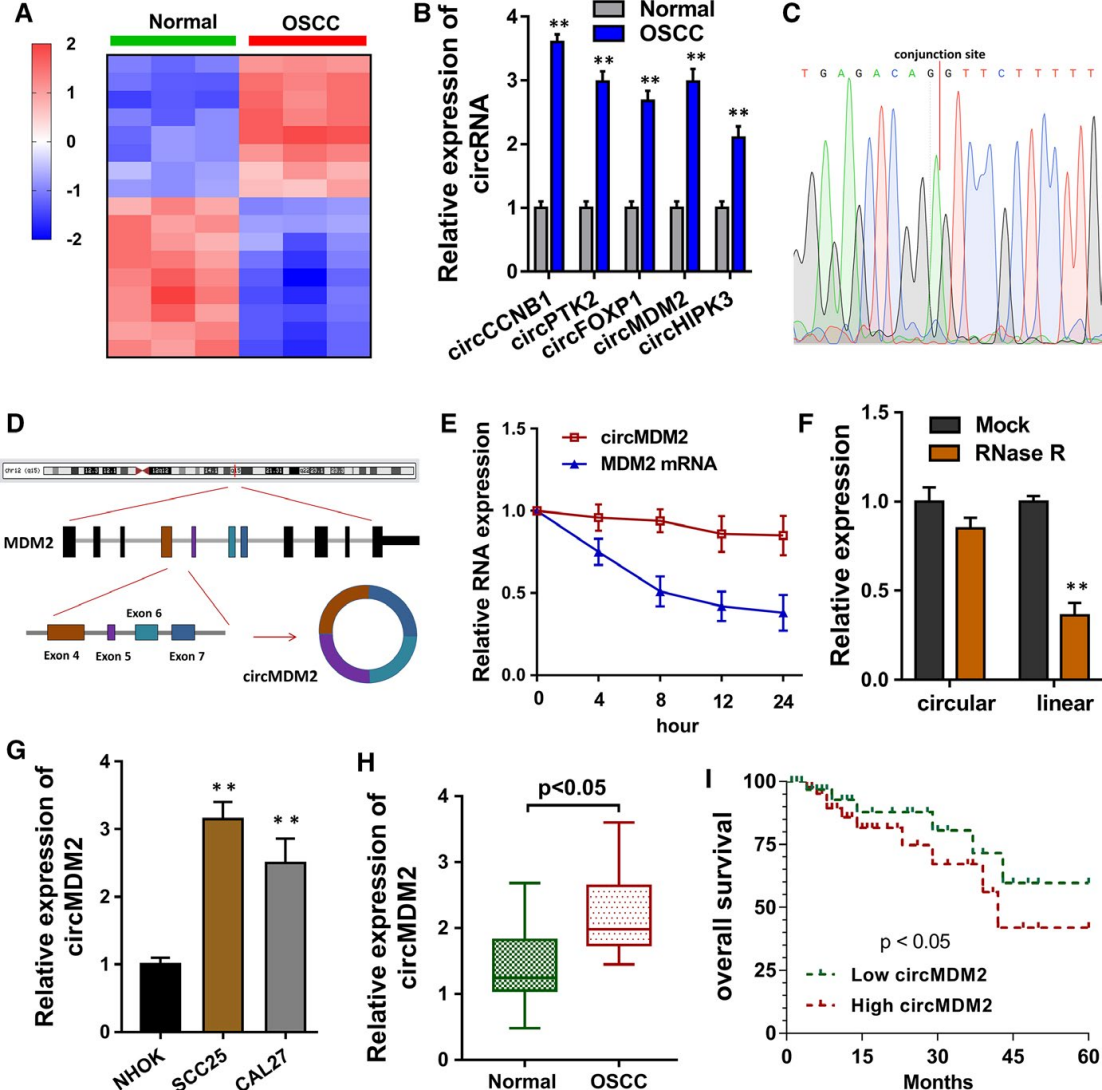
LncRNA circMDM2 indicates the poor prognosis of OSCC patients. A, Heat map for microarray analysis showed the multiple dysregulated circRNAs in the OSCC or normal samples. B, RT‐PCR detected several candidate circRNAs expression in the OSCC specimens as compared to the normal specimens. C, Sanger sequencing revealed the circular loop construction of circMDM2. D, Schematic diagram showed the generation of circMDM2 from exon 7 to exon 4. RT‐PCR detected the circMDM2 expression when treated with (E) actinomycin D and (F) RNase R. G, RT‐PCR detected the circMDM2 level in the OSCC cells (SCC25, CAL27). H, RT‐PCR detected the circMDM2 level in the OSCC specimens as compared to the normal specimens. I, Survival analysis calculated by Kaplan‐Meier plotter indicated the survival rate of OSCC patients with higher or lower circMDM2 levels. **P < .01 vs. control

Microarray analysis found that multiple circRNAs were dysregulated in the OSCC tissue samples (Figure 1A). Besides, we found that several candidate circRNAs, including circMDM2, were consistently up‐regulated in the OSCC specimens as compared to the normal specimens (Figure 1B). Sanger sequencing found that circMDM2 was indeed originated as a circular loop (Figure 1C) and generated from exon 7 to exon 4 (Figure 1D). Actinomycin D administration (Figure 1E) and RNase R administration (Figure 1F) showed that circMDM2 was much more than the linear transcripts. In the OSCC cells (SCC25, CAL27), we found that circMDM2 level was increased in these cells as compared to normal cells (Figure 1G). CircMDM2 expression level was significantly up‐regulated in the OSCC patients' specimens as compared to the normal specimens (Figure 1H). Survival analysis calculated by Kaplan‐Meier plotter indicated that the OSCC patients with higher circMDM2 level had poor survival rate as comparing to who with lower circMDM2 level (Figure 1I). In conclusion, these findings suggested that circRNA circMDM2 indicates the poor prognosis of OSCC patients.

### 3.2 circMDM2 regulates the proliferation of OSCC cells in vitro and in vivo

**FIGURE 2:**
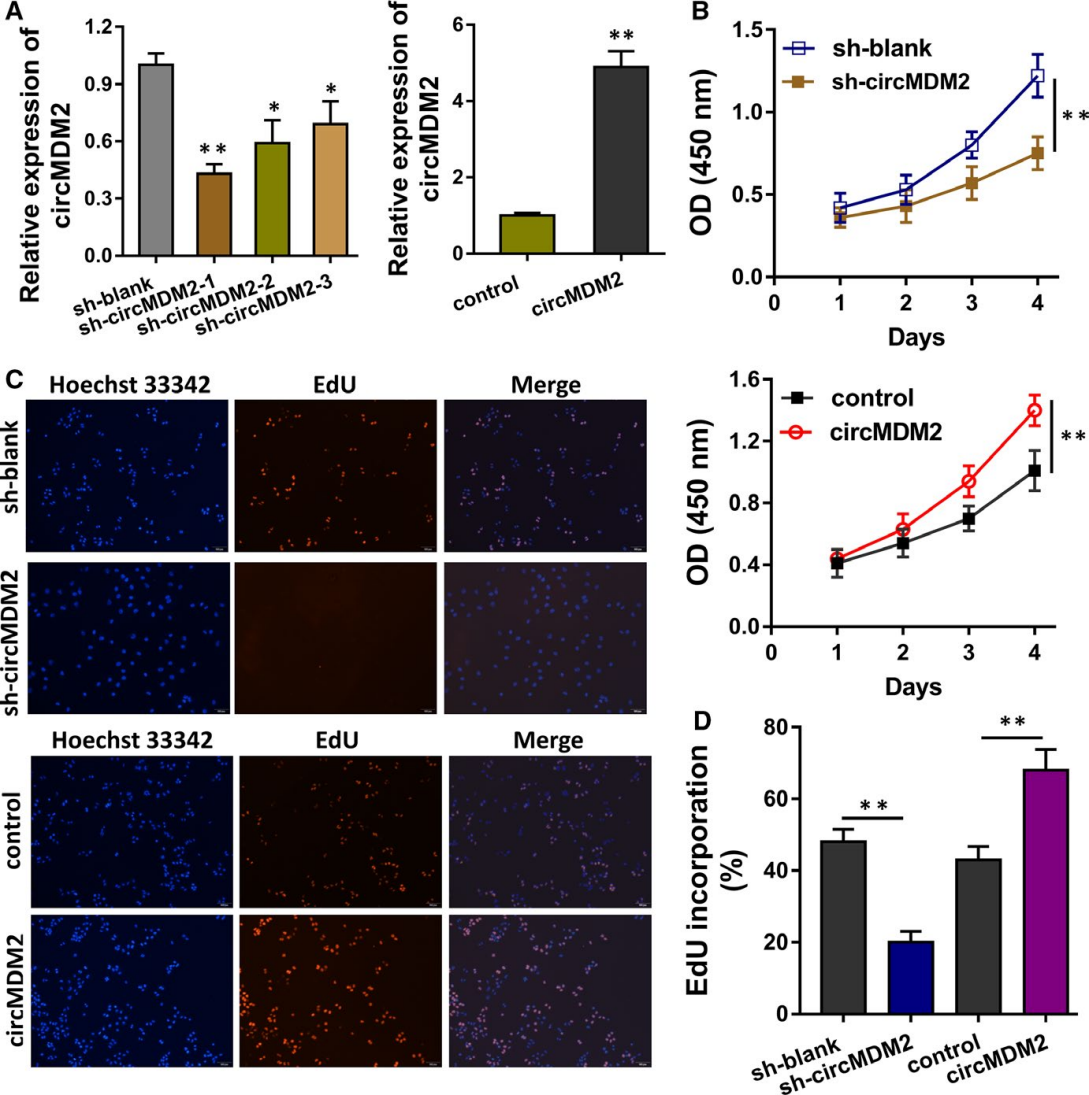
circMDM2 regulates the proliferation of OSCC cells in vitro and in vivo. A, In SCC25 cells, circMDM2 knockdown (sh‐circMDM2) and overexpression (circMDM2) plasmids were transfected. The transfection efficiency was detected using qRT‐PCR. B, CCK‐8 proliferation assays demonstrated the proliferative ability of SCC25 cells transfected with circMDM2 knockdown or circMDM2 overexpression. C and D, EdU assay indicated the proliferative ability of SCC25 cells. E and F, In vivo mice assay indicated the tumour growth in vivo with circMDM2 knockdown injection. *P < .05 vs. control. **P < .01 vs. control

**FIGURE 3:**
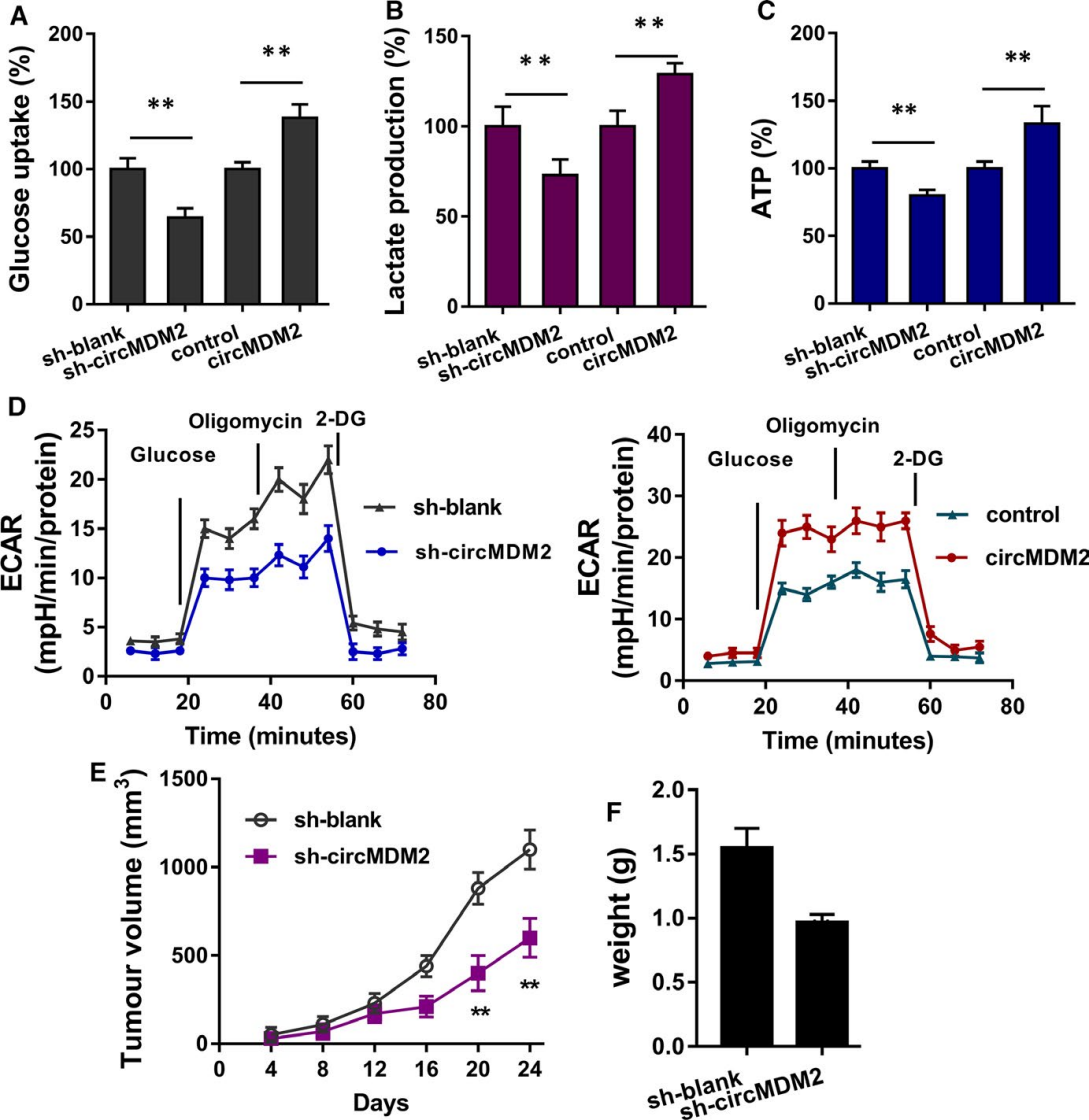
circMDM2 regulates the glycolysis of OSCC cells. A, Glucose uptake analysis illustrated the glucose consumption level in SCC25 cells transfected with circMDM2 knockdown or overexpression. B, Lactate production analysis illustrated the lactate generation level in SCC25 cells transfected with circMDM2 knockdown or overexpression. C, ATP metabolism analysis indicated the ATP generation in SCC25 cells transfected with circMDM2 knockdown or overexpression. D, Extracellular acidification rate (ECAR) assay indicated the glycolytic capacity in SCC25 cells. **P < .01 vs. control

Given that circMDM2 was significantly up‐regulated in the OSCC tissue specimens and cell lines, we assumed that circMDM2 might function as an oncogene in the OSCC tumorigenesis. In the cellular experiments, circMDM2 knockdown and overexpression transfection were constructed in SCC25 cells (Figure 2A). CCK‐8 proliferation assays (Figure 2B) and EdU assay (Figure 2C,D) found that circMDM2 knockdown inhibited the SCC25 cells' proliferative ability and circMDM2 overexpression accelerated the proliferation. In vivo mice assay indicated that circMDM2 knockdown repressed the tumour growth in vivo, indicating that circMDM2 might act as a tumour‐promoting factor in the OSCC origination (Figure 3E,F). Overall, these data indicate that circMDM2 regulates the proliferation of OSCC cells in vitro and in vivo.

### 3.3 circMDM2 regulates the glycolysis of OSCC cells

In addition to the regulation of cellular proliferation mediated by circMDM2, we further investigated the roles on glycolysis. Glucose uptake, lactate production and ATP metabolism analysis indicated that circMDM2 overexpression promoted the glucose uptake, lactate production and ATP level. Moreover, circMDM2 knockdown inhibited the glucose uptake, lactate production and ATP level (Figure 3A‐D). Overall, these data indicate that circMDM2 regulates the glycolysis of OSCC cells.

### 3.4 circMDM2 targets miR‐532‐3p/HK2

**FIGURE 4:**
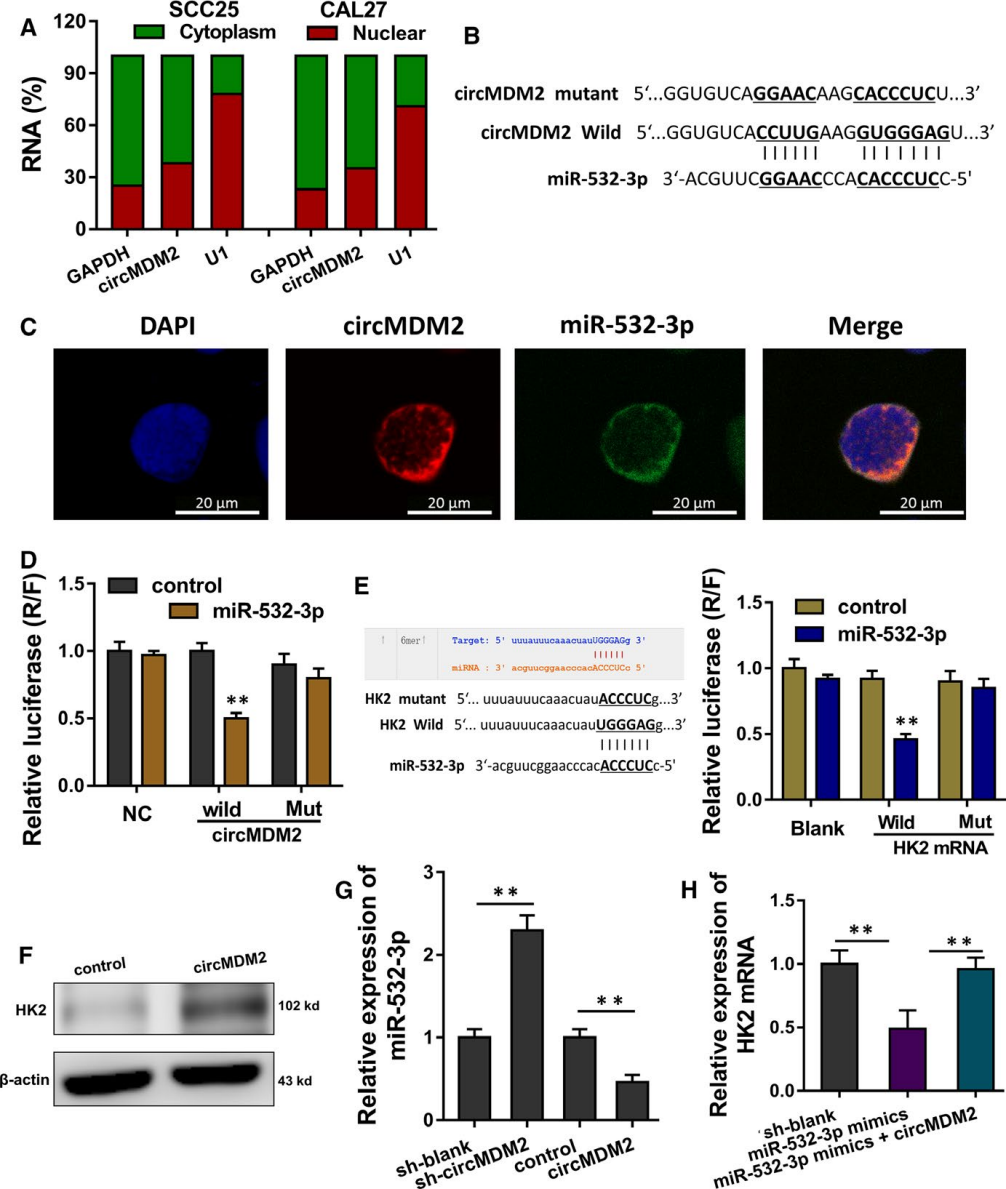
circMDM2 targets miR‐532‐3p/HK2 axis. A, Subcellular fraction analysis illustrated the distribution of circMDM2 in the cytoplasmic or nuclear portion in OSCC cells. B, Online bioinformatics tools (CircInteractome, https://circinteractome.nia.nih.gov) indicated the 3'‐UTR binding of miR‐532‐3p towards circMDM2. C, RNA‐fluorescence in situ hybridization (RNA‐FISH) showed the location of circMDM2 and miR‐532‐3p in SCC25 cells. D, Luciferase reporter assay indicated the interaction of miR‐532‐3p and circMDM2 3'‐UTR wild‐type. E, Online bioinformatics tools (ENCORI, http://starbase.sysu.edu.cn) and luciferase reporter assay indicated the interaction of miR‐532‐3p and the 3'‐UTR of HK2. F, Western blotting analysis indicated the HK2 level in SCC25 cells transfected with circMDM2 overexpression. G, RT‐PCR detected the miR‐532‐3p level in SCC25 cells transfected with circMDM2 knockdown or overexpression. I, RT‐PCR detected the HK2 mRNA level in SCC25 cells transfected with miR‐532‐3p mimics or circMDM2 overexpression. **P < .01 vs. control

In the subcellular fraction analysis, a large proportion of circMDM2 was found to be distributed in the cytoplasmic portion in OSCC cells (Figure 4A). Online bioinformatics tools (CircInteractome, https://circinteractome.nia.nih.gov) indicated that miR‐532‐3p acted as one of the targets of circMDM2 with 3'‐UTR binding (Figure 4B). RNA‐fluorescence in situ hybridization (RNA‐FISH) showed the location of circMDM2 and miR‐532‐3p in SCC25 cells (Figure 4C). Luciferase reporter vectors were constructed for the assays, and results indicated that miR‐532‐3p could interact with circMDM2 3'‐UTR wild‐type (Figure 4D). Moreover, online bioinformatics tools (ENCORI, http://starbase.sysu.edu.cn) indicated that miR‐532‐3p targeted the 3'‐UTR of HK2. Luciferase reporter assay indicated that miR‐532‐3p could interact with HK2 3'‐UTR wild‐type (Figure 4E). Western blotting analysis indicated that circMDM2 overexpression could increase the HK2 level (Figure 4F). RT‐PCR showed that miR‐532‐3p level was up‐ or down‐regulated in SCC25 cells transfected with circMDM2 knockdown or overexpression (Figure 4G). RT‐PCR showed that HK2 mRNA was rescued by the co‐transfection with miR‐532‐3p mimics and circMDM2 overexpression (Figure 4H). Therefore, these data support that circMDM2 targets miR‐532‐3p/HK2 axis.

## 4 DISCUSSION

More and more evidence suggests that ncRNAs, including micro RNA, lncRNA and circular RNA, function as essential regulator in the human cancer origination.^8^, ^17^, ^18^, ^19^ CircRNA could participate in the tumour progression, not only the proliferation, invasion and migration, but also the metastasis and recurrence. The known and unknown functions of circRNAs need more attention in future.

In present research, we found that circMDM2 was significantly up‐regulated in the OSCC cells. Clinically, the high expression of circMDM2 indicated the poor prognosis of OSCC individuals in large cohort research. In the following experimental assays, knockdown and overexpression transfection for circMDM2 were both constructed in SCC25 cells. Gain‐ and loss‐of‐function experiments showed that circMDM2 promoted that proliferation of OSCC cells. Interestingly, we found that circMDM2 could promote the glucose consumption, lactate production and ATP level, suggesting the promotion of circMDM2 for OSCC glycolysis.

In human cancer, circRNAs have been found to be critical regulators in the OSCC tumorigenesis. For example, circATRNL1 is significantly down‐regulated in OSCC tissue and closely related to tumour progression, and up‐regulation of circATRNL1 enhanced the radiosensitivity of OSCC by directly binding to miR‐23a‐3p to relieve inhibition for PTEN.^20^ Another down‐regulated circ_0000140 is derived from exons 7 to 10 of KIAA0907 gene, and circ_0000140 binds with miR‐31 to up‐regulate the target gene LATS2 to regulate OSCC cellular EMT.^21^ CircRNA_100290 and GLUT1 are remarkably increased in OSCC tissue specimens and cells, and circRNA_100290/miR‐378a/GLUT1 axis promotes the proliferation and glycolysis in OSCC cell lines.^22^ Therefore, we found that circRNA congruously acted as the oncogene in OSCC.

In the subcellular location of OSCC cells, we found that circMDM2 was mainly located in the cytoplasmic segment, suggesting the potential post‐transcriptional regulation for circMDM2. Besides, in the previously finding, we found that HK2 and circMDM2 were positively correlated; thus, we came up with an assumption that circMDM2 was likely to interact with HK2 through a connector by indirect manner. Finally, with the help of bioinformatics prediction tools and luciferase reporter assays, we found that miR‐532‐3p functioned as the bridge of circMDM2 and HK2, forming the circMDM2/miR‐532‐3p/HK2 axis.

The metabolic reprogramming phenomenon in cancer is known as the Warburg effect, a significant hallmark for cancer energy metabolic system.^23^ HK2 is an essential oncogene in human cancer and modulate the glycolysis in cancer. For example, Tan VP et al found that HK2 functions as a switch from glycolysis to ensure cellular energy homeostasis at starvation condition.^24^ Besides, miR‐532‐3p was identified to be an anti‐oncogene in human cancer, including tongue squamous cell carcinoma^25^ and colorectal cancer.^26^

In conclusion, these findings demonstrated that circMDM2 promoted the proliferation and glycolysis of OSCC through circMDM2/miR‐532‐3p/HK2 axis. Therefore, circMDM2 may be a novel and promising therapeutic sensitizer for OSCC cells.

## CONFLICT OF INTEREST

All authors declare no conflicts of interest.

## AUTHORS' CONTRIBUTION

Zhao Zheng performed these experiments. Xiaozhou Ma and Hongfa Li acted as the assistant.

## Supporting information

Table S1

## Data Availability

Data available on request from the authors.

## References

[1] Jayaraj R , Kumarasamy C , Madurantakam Royam M , et al. Letter to the editor: is HIF‐1alpha a viable prognostic indicator in OSCC? A critical review of a meta‐analysis study. World J Surg Oncol. 2018;16:111. 29914529 10.1186/s12957-018-1408-4 PMC6006741

[2] Kang MK , Chen W , Park NH . Regulation of epithelial cell proliferation, differentiation, and plasticity by grainyhead‐like 2 during oral carcinogenesis. Crit Rev Oncog. 2018;23:201‐217. 30311575 10.1615/CritRevOncog.2018027608

[3] Pollaers K , Hinton‐Bayre A , Friedland PL , et al. AJCC 8th Edition oral cavity squamous cell carcinoma staging—Is it an improvement on the AJCC 7th edition? Oral Oncol. 2018;82:23‐28. 29909897 10.1016/j.oraloncology.2018.04.018

[4] Ramos‐Garcia P , Gonzalez‐Moles MA , Gonzalez‐Ruiz L , et al. Prognostic and clinicopathological significance of cyclin D1 expression in oral squamous cell carcinoma: a systematic review and meta‐analysis. Oral Oncol. 2018;83:96‐106. 30098785 10.1016/j.oraloncology.2018.06.007

[5] Sciubba JJ , Larian B . Oral squamous cell carcinoma: early detection and improved 5‐year survival in 102 patients. Gen Dent. 2018;66:e11‐e16. 30444715

[6] Vasileios SP , Kyrodimos E , Tsiambas E , et al. Chromosomal instability in oral squamous cell carcinoma. J Balk Union Oncol. 2018;23:1580‐1582. 30610780

[7] Zhao W , Chu S , Jiao Y . Present scenario of circular RNAs (circRNAs) in plants. Front Plant Sci. 2019;10:379. 31001302 10.3389/fpls.2019.00379 PMC6454147

[8] Wu J , Qi X , Liu L , et al. Emerging epigenetic regulation of circular RNAs in human cancer. Mol Ther‐Nucl Acids. 2019;16:589‐596. 10.1016/j.omtn.2019.04.011 PMC6517616 31082792

[9] Zhou C , Liu H‐S , Wang F‐W , et al. circCAMSAP1 promotes tumor growth in colorectal cancer via the miR‐328‐5p/E2F1 axis. Mol Ther. 2020;28(3):914‐928. 31951832 10.1016/j.ymthe.2019.12.008 PMC7054739

[10] Wang M , Jiang S , Wu W , et al. Non‐coding RNAs function as immune regulators in teleost fish. Non‐coding RNA. 2018;9:2801. 10.3389/fimmu.2018.02801 PMC6279911 30546368

[11] Ji F , Du R , Chen T , et al. Circular RNA circSLC26A4 accelerates cervical cancer progression via miR‐1287‐5p/HOXA7 axis. Mol Ther‐Nucl Acids. 2019;19:413‐420. 10.1016/j.omtn.2019.11.032 PMC6940609 31896069

[12] Sun X , Wang M , Xu R , et al. Prognostic model based on circular RNA circPDK1 for resected lung squamous cell carcinoma. Transl Lung Cancer Res. 2019;8:907‐919. 32010569 10.21037/tlcr.2019.11.20 PMC6976365

[13] Wang X , Cao X , Dong D , et al. Circular RNA TTN Acts as a miR‐432 sponge to facilitate proliferation and differentiation of myoblasts via the IGF2/PI3K/AKT signaling pathway. Mol Ther‐Nucl Acids. 2019;18:966‐980. 10.1016/j.omtn.2019.10.019 PMC6881651 31770673

[14] Dou Z , Li S , Ren W , et al. Decreased expression of hsa_circ_0072387 as a valuable predictor for oral squamous cell carcinoma. Oral Dis. 2019;25:1302‐1308. 30908839 10.1111/odi.13094

[15] Su W , Wang Y , Wang F , et al. Hsa_circ_0005379 regulates malignant behavior of oral squamous cell carcinoma through the EGFR pathway. BMC Cancer. 2019;19:400. 31035951 10.1186/s12885-019-5593-5 PMC6489207

[16] Chen Z , Zuo X , Zhang Y , et al. MiR‐3662 suppresses hepatocellular carcinoma growth through inhibition of HIF‐1alpha‐mediated Warburg effect. Cell Death Dis. 2018;9:549. 29748591 10.1038/s41419-018-0616-8 PMC5945826

[17] Zhao W , Cui Y , Liu L , et al. Splicing factor derived circular RNA circUHRF1 accelerates oral squamous cell carcinoma tumorigenesis via feedback loop. Cell Death Differ. 2020;27:919‐933. 31570856 10.1038/s41418-019-0423-5 PMC7206121

[18] Zhao W , Qi X , Liu L , et al. Epigenetic regulation of m6A modifications in human cancer. Mol Ther‐Nucl Acids. 2019;19:405‐412. 10.1016/j.omtn.2019.11.022 PMC6938965 31887551

[19] Yang J , Liu J , Zhao S , et al. N6‐Methyladenosine METTL3 modulates the proliferation and apoptosis of lens epithelial cells in diabetic cataract. Mol Ther‐Nucl Acids. 2020;20:111‐116. 10.1016/j.omtn.2020.02.002 PMC7066033 32163892

[20] Chen G , Li Y , He YI , et al. Upregulation of circular RNA circATRNL1 to sensitize oral squamous cell carcinoma to irradiation. Mol Ther‐Nucl Acids. 2020;19:961‐973. 10.1016/j.omtn.2019.12.031 PMC7005496 32032888

[21] Peng QS , Cheng YN , Zhang WB , et al. circRNA_0000140 suppresses oral squamous cell carcinoma growth and metastasis by targeting miR‐31 to inhibit Hippo signaling pathway. Cell Death Dis. 2020;11:112. 32041942 10.1038/s41419-020-2273-y PMC7010827

[22] Chen X , Yu J , Tian H , et al. Circle RNA hsa_circRNA_100290 serves as a ceRNA for miR‐378a to regulate oral squamous cell carcinoma cells growth via Glucose transporter‐1 (GLUT1) and glycolysis. J Cell Physiol. 2019;234:19130‐19140. 31187488 10.1002/jcp.28692

[23] Jing Y‐Y , Cai F‐F , Zhang L , et al. Epigenetic regulation of the Warburg effect by H2B monoubiquitination. Cell Death Differ. 2019. 10.1038/s41418-019-0450-2 PMC7206070 31685978

[24] Tan VP , Miyamoto S . HK2/hexokinase‐II integrates glycolysis and autophagy to confer cellular protection. Autophagy. 2015;11:963‐964. 26075878 10.1080/15548627.2015.1042195 PMC4502787

[25] Feng C , So HI , Yin S , et al. MicroRNA‐532‐3p suppresses malignant behaviors of tongue squamous cell carcinoma via regulating CCR7. Front Pharmacol. 2019;10:940. 31555130 10.3389/fphar.2019.00940 PMC6727182

[26] Gu C , Cai J , Xu Z , et al. MiR‐532‐3p suppresses colorectal cancer progression by disrupting the ETS1/TGM2 axis‐mediated Wnt/beta‐catenin signaling. Cell Death Dis. 2019;10:739. 31570702 10.1038/s41419-019-1962-x PMC6768886

