# Supplementary material for: Circular RNA circMDM2 accelerates the glycolysis of oral squamous cell carcinoma by targeting miR‐532‐3p/HK2
Source: J Cell Mol Med. 2020 May 15;24(13):7531–7. doi: 10.1111/jcmm.15380 (PMC7339225; doi:10.1111/jcmm.15380)
Supplement: Supplementary file 1 — Table S1 [file JCMM-24-7531-s001.docx]

**Table S1.** Primers sequences for qRT-PCR and sequences of shRNA.

|  | Sequences |
| --- | --- |
| circMDM2 | forward 5’-AGAGCTTCAGGAAGAGAAACCT-3’  reverse 5’-ACAATATGTTGTTGCTTCTCATCA-3’ |
| sh-circMDM2-1 | GTGAGACAGGTTCTTTTTTAT |
| sh-circMDM2-2 | GAGACAGGTTCTTTTTTATCT |
| sh-circMDM2-3 | ATTAGTGAGACAGGTTCTTTT |
| HK2 | forward, 5’-GAGCCACCACTCACCCTACT-3’  reverse, 5’-CCAGGCATTCGGCAATGTG-3’ |
| miR-532-3p | 5’-ATCCTCCCACACCCAAGG-3’ |
| U6 | forward, 5’-CTCGCTTCGGCAGCACA-3’  reverse, 5’-CACAGCTTCTCTTTGATGTCAC-3’ |
| beta-actin | forward 5’-CTCCATCCTGGCCTCGCTGT-3’  reverse 5’-GCTGTCACCTTCACCGTTCC-3’ |
